# Efficacy of Integrated Risk Score Using Omics-Based Biomarkers for the Prediction of Acute Rejection in Kidney Transplantation: A Randomized Prospective Pilot Study

**DOI:** 10.3390/ijms25105139

**Published:** 2024-05-09

**Authors:** Jeong-Hoon Lim, Byung Ha Chung, Sang-Ho Lee, Jong Soo Lee, Yeong Hoon Kim, Man-Hoon Han, Hee-Yeon Jung, Ji-Young Choi, Jang-Hee Cho, Sun-Hee Park, Yong-Lim Kim, Chan-Duck Kim

**Affiliations:** 1 Division of Nephrology, Department of Internal Medicine, School of Medicine, Kyungpook National University, Kyungpook National University Hospital, Daegu 41944, Republic of Korea; jh-lim@knu.ac.kr (J.-H.L.); hy-jung@knu.ac.kr (H.-Y.J.); jyss1002@hanmail.net (J.-Y.C.); jh-cho@knu.ac.kr (J.-H.C.); sh-park@knu.ac.kr (S.-H.P.); ylkim@knu.ac.kr (Y.-L.K.); 2 Division of Nephrology, Department of Internal Medicine, Seoul St. Mary’s Hospital, College of Medicine, The Catholic University of Korea, Seoul 06591, Republic of Korea; chungbh@catholic.ac.kr; 3 Division of Nephrology, Department of Internal Medicine, College of Medicine, Kyung Hee University, Seoul 02447, Republic of Korea; lshkidney@khu.ac.kr; 4 Division of Nephrology, Department of Internal Medicine, University of Ulsan College of Medicine, Ulsan 44033, Republic of Korea; 5 Division of Nephrology, Department of Internal Medicine, College of Medicine, Inje University Busan Paik Hospital, Busan 47392, Republic of Korea; yeonghnl@inje.ac.kr; 6 Department of Pathology, School of Medicine, Kyungpook National University, Kyungpook National University Hospital, Daegu 41944, Republic of Korea; mhhan1@knu.ac.kr

**Keywords:** biomarker, graft rejection, kidney transplantation, omics

## Abstract

Acute rejection (AR) is critical for long-term graft survival in kidney transplant recipients (KTRs). This study aimed to evaluate the efficacy of the integrated risk score of omics-based biomarkers in predicting AR in KTRs. This prospective, randomized, controlled, multicenter, pilot study enrolled 40 patients who recently underwent high-immunologic-risk kidney transplantation (KT). Five omics biomarkers were measured, namely, blood mRNA (three-gene signature), urinary exosomal miRNA (three-gene signature), urinary mRNA (six-gene signature), and two urinary exosomal proteins (hemopexin and tetraspanin-1) at 2 weeks and every 4 weeks after KT for 1 year. An integrated risk score was generated by summing each biomarker up. The biomarker group was informed about the integrated risk scores and used to adjust immunosuppression, but not the control group. The outcomes were graft function and frequency of graft biopsy. Sixteen patients in the biomarker group and nineteen in the control group completed the study. The mean estimated glomerular filtration rate after KT did not differ between the groups. Graft biopsy was performed in two patients (12.5%) and nine (47.4%) in the biomarker and control groups, respectively, with the proportion being significantly lower in the biomarker group (*p* = 0.027). One patient (6.3%) in the biomarker group and two (10.5%) in the control group were diagnosed with AR, and the AR incidence did not differ between the groups. The tacrolimus trough level was significantly lower in the biomarker group than in the control group at 1 year after KT (*p* = 0.006). Integrated omics biomarker monitoring may help prevent unnecessary or high-complication-risk biopsy and enables tailored immunosuppression by predicting the risk of AR in KTRs.

## 1. Introduction

Kidney transplantation (KT) is the best treatment option for patients with end-stage kidney disease [1,2]. It has been shown to improve patients’ survival and quality of life compared with dialysis [3,4]. Furthermore, with the advances in various immunosuppressive therapies, the long-term outcome of KT is steadily improving [5].

However, acute rejection (AR) remains the most important complication that impairs graft function in kidney transplant recipients (KTRs) [6,7]. Although many attempts have been made to diagnose AR early, the only definitive diagnostic method for AR is graft biopsy.

Graft biopsy has several drawbacks, such as the risk of bleeding or infection and difficulty of serial follow-up [7,8]. To overcome these drawbacks, much attention has been paid to the development of noninvasive biomarkers for the early diagnosis and prediction of AR. Over the past two decades, omics-based immunologic monitoring and rejection prediction techniques for KTRs have gained attention [9]. Our group has discovered the utility of serum or urine mRNA, miRNA, and proteins for the early diagnosis of AR using omics technology [7,10,11,12,13]. The effects of these biomarkers were validated in our previous studies, but the interpretation of the results for the effects of biomarkers is limited. We utilized retrospective cross-sectional data and did not evaluate the efficacy of integrated omics-based biomarkers.

Therefore, to overcome the limitations of previous studies, this prospective clinical study aimed to evaluate the clinical efficacy of the integrated risk score of omics-based biomarkers in predicting AR in KTRs with high immunologic risk.

## 2. Results

### 2.1. Baseline Characteristics

Of the 40 patients enrolled in the study, 5 withdrew their consent, whereas 16 and 19 KTRs in the biomarker and control groups, respectively, completed the study (Supplementary Figure S1). The patients’ baseline characteristics are presented in Table 1. For the biomarker group, the mean age of the patients was 49.4 ± 11.0 years, and 75.0% of them were men. For the control group, the mean age of the patients was 52.3 ± 13.4 years, and 68.4% of them were men. Baseline demographics, such as age, sex, body mass index, primary renal disease, and comorbidities, did not differ between the groups. Information on immunologic risk, such as human leukocyte antigen (HLA) mismatch numbers, direct cross-match positive ratio, and type of induction immunosuppressant used, also did not differ between the groups. The frequency of pretransplant desensitization was also not different between the biomarker and control groups (81.3% vs. 73.7%, *p* = 0.595). Graft function at the time of discharge after KT was similar between the groups.

### 2.2. Clinical Information at 1 Year after KT

Clinical information at 1 year after KT is presented in Table 2. The primary outcome, estimated glomerular filtration rate (eGFR) at 1 year, did not differ between the groups. Changes in serum creatinine levels at baseline and at 1 year also did not differ between the groups. The proportion of patients with serum creatinine over 1.5 mg/dL at 1 year tended to be lower in the biomarker group than in the control group (*p* = 0.062).

The biomarker group had a lower proportion of patients who underwent graft biopsy at 1 year after KT than the control group (2 [12.5%] vs. 9 [47.4%], *p* = 0.027). Of the two biopsies in the biomarker group, one was performed for decreased eGFR and one for increased proteinuria; of the nine biopsies in the control group, six were performed for decreased eGFR, one for increased proteinuria, and two for suspected BK virus-associated nephropathy. The incidence of AR was low in both groups (one T-cell-mediated rejection [TCMR] [6.3%] in the biomarker group and two ABMR [10.5%] in the control group), and there was no difference between the groups. One patient in the biomarker group (6.3%) and two patients (10.5%) in the control group developed de novo donor-specific antibody over 1 year, with no difference in incidence between the groups. There was no difference in the incidence of BK viremia/viruria (biomarker vs. control: 6.3% vs. 10.5%) and acute calcineurin inhibitor nephrotoxicity (biomarker vs. control: 0% vs. 10.5%) between the two groups.

### 2.3. Changes in the Integrated Risk Score and Each Biomarker at 1 Year after KT

**Figure 1:**
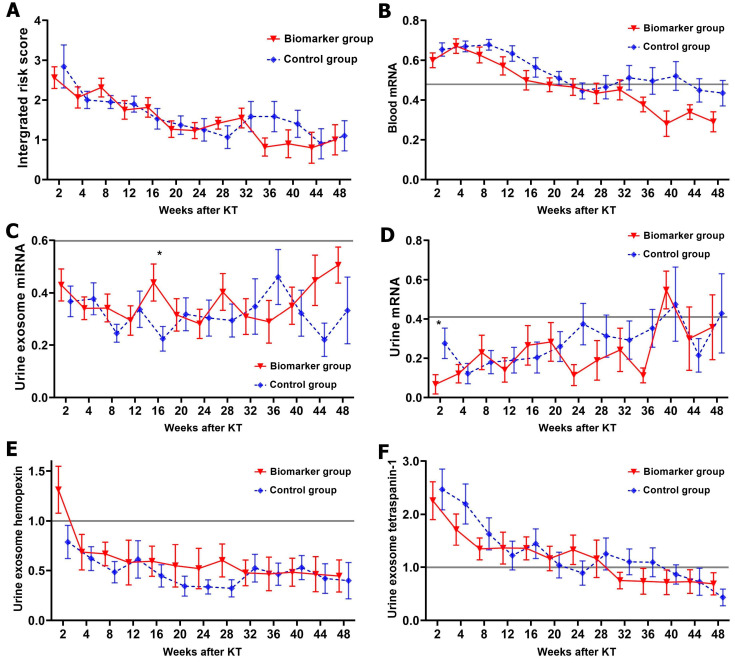
Serial changes of integrated risk score and each biomarker. (**A**) Integrated risk score. (**B**) Blood mRNA for 3 genes (*ARG1*, *MAPK9*, and *OLR1*). (**C**) Urine exosomal miRNA for 3 genes (*miR-21-5p*, *miR-31-5p*, and *miR-4532*). (**D**) Urine mRNA for 6 genes (*C1QB*, *CD3e*, *CXCL-9*, *IP-10*, *PSMB9*, and *Tim-3*). (**E**) Urine exosomal hemopexin. (**F**) Urine exosomal tetraspanin-1. The gray solid line indicates the cutoff value. * indicates *p* < 0.05. Abbreviation: KT, kidney transplantation.

The mean integrated risk scores at 2 weeks after KT were 2.6 ± 1.1 and 2.8 ± 2.4 in the biomarker and control groups, respectively (Figure 1A). During the observational period, the integrated risk score gradually decreased after KT, and after 1 year, it was 1.0 ± 1.4 in the biomarker group and 1.1 ± 1.2 in the control group. No significant difference was observed in the integrated risk scores between the groups during the follow-up period. Figure 1B–F present the changes in each omics biomarker. No difference was observed in most biomarker measurements between the groups.

### 2.4. Changes in Graft Function and Tacrolimus trough Level 1 Year after KT

**Figure 2:**
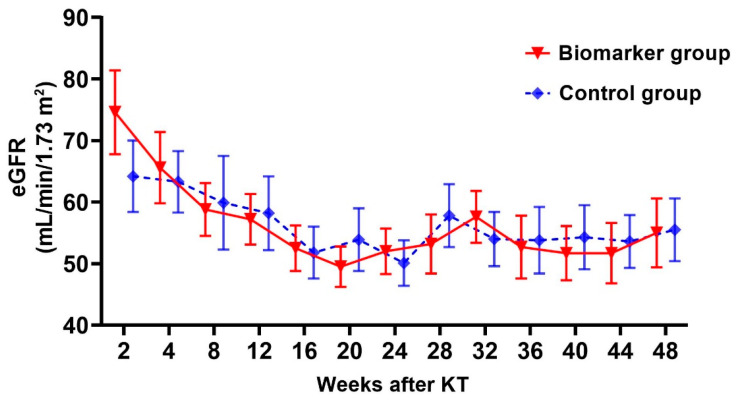
Serial change in eGFR. Abbreviations: eGFR, estimated glomerular filtration rate; KT, kidney transplantation.

Serial changes in eGFR at 1 year after KT are presented in Figure 2. The mean eGFRs at 2 weeks after KT were 74.7 ± 27.6 mL/min/m^2^ in the biomarker group and 64.3 ± 25.5 mL/min/m^2^ in the control group (*p* = 0.256). The eGFR of both groups remained stable, and no significant difference was observed at any time point.

**Figure 3:**
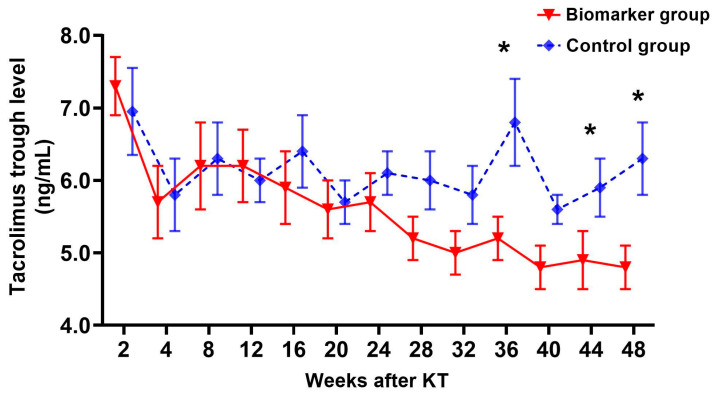
Serial change in the tacrolimus trough level. * indicates *p* < 0.05. Abbreviation: KT, kidney transplantation.

Changes in the tacrolimus trough level 1 year after KT are presented in Figure 3. The mean tacrolimus trough levels at 2 weeks after KT were 7.3 ± 1.8 ng/mL in the biomarker group and 7.0 ± 2.8 ng/mL in the control group (*p* = 0.695). The tacrolimus trough level did not differ at 32 weeks after KT. However, from 36 to 48 weeks, the tacrolimus trough level in the biomarker group was significantly lower than that in the control group (*p* < 0.05).

### 2.5. Changes in the Integrated Risk Score and Histopathologic Findings of KTRs with AR

**Figure 4:**
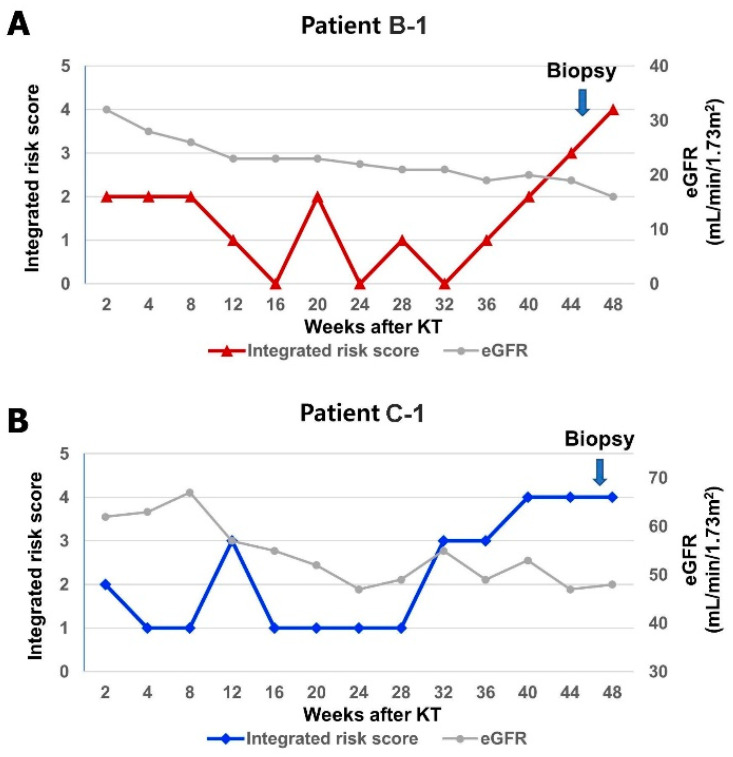

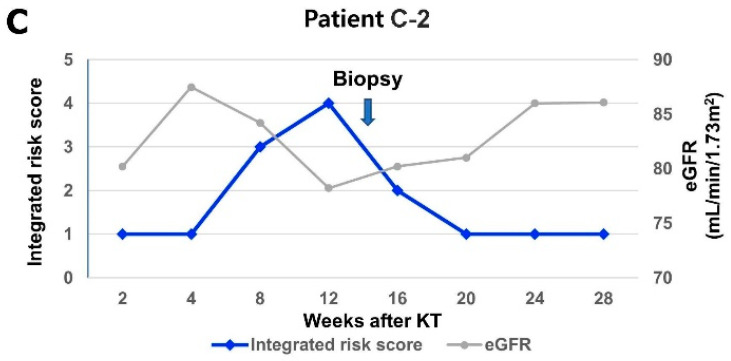
Serial change in integrated risk score and eGFR in KTRs with acute rejection. (**A**) Patient in the biomarker group with acute TCMR (B-1). Biomarker scores at biopsy: Integrated risk score: 3; blood mRNA: 0; urine exosomal miRNA: 0; urine mRNA: 1; urine exosomal hemopexin: 1; urine exosomal tetraspanin-1: 1. (**B**,**C**) Patients in the control group with active ABMR (C-1 and C-2). Biomarker scores at biopsy: C-1: integrated risk score: 4; blood mRNA: 1; urine exosomal miRNA: 0; urine mRNA: 1; urine exosomal hemopexin: 1; urine exosomal tetraspanin-1: 1; C-2: integrated risk score: 4; blood mRNA: 1; urine exosomal miRNA: 0; urine mRNA: 1; urine exosomal hemopexin: 1; urine exosomal tetraspanin-1: 1. Abbreviations: eGFR, estimated glomerular filtration rate; KT, kidney transplantation.

**Figure 5:**
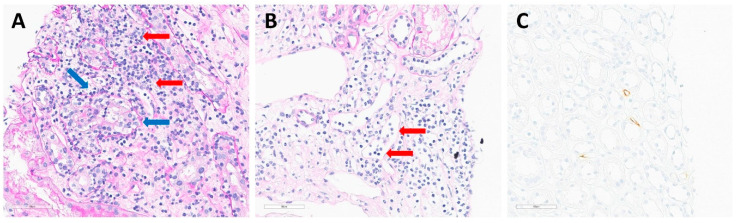
Representative histopathological findings in acute rejection. (**A**) There is a moderate degree of interstitial inflammation (red arrows; i2) with severe lymphocytic tubulitis (blue arrows; t3) (periodic acid-Schiff, ×400). These findings are compatible with acute T-cell mediated rejection. (**B**) There is moderate peritubular capillaritis (red arrows; ptc2) (periodic acid-Schiff, ×400) and (**C**) C4d positivity was observed in peritubular capillaries (brown color). Less than 10% of peritubular capillaries stained, indicating C4d1 (immunohistochemistry for C4d, ×400). These findings are consistent with active antibody-mediated rejection.

Three KTRs were diagnosed with AR; the serial changes in their integrated risk score and eGFR are presented in Figure 4. In one patient in the biomarker group (B-1), the integrated risk score remained low until 32 weeks but continued to increase from 36 weeks (Figure 4A). The eGFR did not significantly decrease, whereas the risk score increased. The patient was diagnosed with acute TCMR, IB (Banff lesion score: tubulitis [t]3, interstitial inflammation [i]2, glomerulitis [g]1, peritubular capillaritis [ptc]2, microvascular inflammation [MVI]3) (Table 3). Figure 5A shows severe lymphocytic tubulitis with moderate interstitial inflammation in the patient. In one patient in the control group (C-1), the integrated risk score remained low until 28 weeks but continued to increase from 32 weeks (Figure 4B). A physician was not informed of the change in the integrated risk score as the patient belonged to the control group. Graft biopsy was performed based on conventional indices, such as a decrease in graft function. The patient was diagnosed with active ABMR (Banff lesion score: g0, ptc2, MVI2, C4d1) (Table 3). Figure 5B shows a moderate degree of peritubular capillary inflammation, and Figure 5C shows positive C4d staining by immunohistochemistry in the patient. In another patient in the control group (C-2), the integrated risk score remained low until 4 weeks but increased from 8 weeks with a decrease in eGFR (Figure 4C). The patient was diagnosed with active ABMR (Banff lesion score: t1, i2, g2, ptc2, MVI4, C4d1) (Table 3).

## 3. Discussion

To the best of our knowledge, this study is the first prospective clinical trial to investigate the efficacy of the integrated risk score of omics biomarkers in KTRs. The integrated risk score of five different omics biomarkers was the highest in the early period of KT and decreased over time. The biomarker group used an integrated risk score to evaluate the risk of AR, and no difference was observed between graft function and the incidence of AR at the 1 year follow-up. However, the biomarker group showed modulated intensity of immunosuppression and a reduction in graft biopsy frequency without increases in AR incidence. These results indicate that the use of the integrated risk score of omics biomarker might help reduce biopsy-related and infectious complications and maintain graft function.

Recent advances in molecular biology have allowed for high-resolution sequence mapping of structural variation in humans [9,14,15]. Omics technology is based on a holistic view of the molecules that constitute an individual; it has accelerated the discovery of biomarkers in transplantation [9,15,16]. In previous studies, we developed five omics biomarkers that predict rejection using transcriptomics and proteomics [7,10,11,12,13]. Each of these biomarkers exhibited efficacy in predicting rejection in a cross-sectional study; however, the predictive power for AR using a single biomarker is limited by the heterogeneous status of AR. To overcome this limitation, we used an integrated risk score to maximize the predictive potential of omics biomarkers for AR. Biomarker studies have traditionally relied on retrospective data for both discovery and validation studies, which has been identified as a significant limitation [17]. This study enhanced the generalizability of omics biomarkers by confirming the clinical efficacy of retrospectively verified omics-based biomarkers in a prospective clinical study.

In the biomarker and control groups, each biomarker showed a similar trend of change in the first year, with blood mRNA and urinary exosomal proteomics biomarkers (hemopexin and tetraspanin-1) initially increasing after KT and then gradually decreasing to below the cutoff. On the other hand, the urinary mRNA biomarker was low initially after KT and gradually increased with time after KT, suggesting that more long-term observational studies are needed. With the changing trend of these biomarkers, the mean of the integrated risk score was also high in the early post-KT period and then gradually decreased. The mean of the integrated risk score was above 2 in the first 2 weeks after KT, which suggests that the integrated risk score is probably not applicable in the acute phase of KT and may be applicable in the subacute phase. In addition, the threshold for the integrated biomarker risk score has not yet been established, so in this study, we increased the intensity of immunosuppression with each one-point increase. The timing and threshold values for the application of the integrated risk score require further study.

In KTRs diagnosed with AR, the integrated risk score decreased early after KT and then gradually increased 2 to 3 months before the AR diagnosis. At the time of biopsy, the integrated risk score was significantly higher, which was 3 or 4. Thus, if the integrated risk score drops and then rises above 3, the risk of AR needs to be considered. In addition, as there was no significant change in graft function in the early stages of the increase in the integrated risk score, it is expected that adjustment of immunosuppressive drugs through biomarker monitoring will help prevent the development of AR. However, in this study, surveillance protocol biopsy was not performed due to complication risks and patient discomfort; therefore, the exact histopathologic findings, such as the presence of subclinical rejection at the early stage of integrated risk score increase, were unknown. These will need to be confirmed in further follow-up studies.

Graft biopsy is a useful tool for identifying and differentiating immunologic or nonimmunologic causes of graft dysfunction [18]. However, the application of graft biopsy is limited by several factors such as bleeding risk, cost, and patient inconvenience; thus, it is mainly performed only for acute graft dysfunction. Protocol biopsy for diagnosing subclinical rejection is not generally performed despite its reported advantages [18,19]. This study demonstrates that biomarker monitoring can significantly reduce the frequency of graft biopsy. The clinical decision to perform graft biopsy is not easy for transplant clinicians; however, the use of biomarkers in addition to traditional indicators can help clinicians in making decisions. A reduction in unnecessary or contraindicated graft biopsies using a biomarker monitoring system will help reduce biopsy-related complications and improve patients’ quality of life.

Another advantage of biomarkers is their ability to facilitate the adjustment of immunosuppressant dosage. Excessive use of immunosuppressants reduces the occurrence of rejection but increases the risk of complications such as BK virus nephropathy or other infectious diseases [20,21,22,23]. Therefore, balanced immunosuppression that considers both the risk of rejection and complications is imperative. In this study, we demonstrated that individualized immunosuppression with biomarker monitoring has the potential to maintain a low intensity of immunosuppression that helps prevent infection without increasing the risk of rejection. This needs to be validated in future large-scale studies.

Omics biomarkers are the cornerstone of precision medicine [9,24]. They integrate traditional clinical information and tailor medical care to choose the optimal treatment options for each KTR. In this study, we confirmed the efficacy of the omics biomarker monitoring system, but the cost-effectiveness, which is one of the most important factors for biomarkers, could not be confirmed. This should be confirmed in future long-term prospective studies.

This study has several limitations. First, the sample size was too small and the follow-up duration was too short to clearly confirm the efficacy of the integrated risk score. Second, although patients had a high-immunologic risk, the incidence of AR was low during the study period. Third, because no previous studies have compared the predictive power of AR among biomarkers, we arbitrarily assigned each omics biomarker an unweighted score of 1 if it exceeded the cutoff and summed them to calculate an integrative risk score. Although this calculation method has no reference, we applied it because no previous studies have combined biomarkers. The results of this pilot study will serve as a basis for future integrative omics biomarker studies.

Monitoring of the integrated risk score using five omics biomarkers reduced the frequency of graft biopsy and immunosuppression. Furthermore, AR incidence and graft function did not differ between the biomarker and control groups. Integrated omics biomarker monitoring may allow for tailored immunosuppression by predicting the risk of AR in KTRs and preventing unnecessary or high-complication-risk biopsy.

## 4. Materials and Methods

### 4.1. Study Design and Participants

This is a prospective, randomized, controlled, multicenter, pilot study conducted to evaluate the efficacy of biomarkers for the early diagnosis and prediction of AR in de novo KTRs (ACROBIOMARKER trial). The study enrolled patients from five tertiary hospitals in Korea (Kyungpook National University Hospital, Kyung Hee University Hospital at Gangdong, the Catholic University of Korea Seoul St. Mary’s Hospital, Inje University Busan Paik Hospital, and Ulsan University Hospital). The detailed inclusion and exclusion criteria are provided at the Clinical Research Information Service (CRiS) at the Korea Centers for Disease Control and Prevention (http://cris.nih.go.kr; accessed on 1 April 2024). In brief, 40 adult patients who underwent high-immunologic-risk KT and maintained stable graft function at 2 weeks following KT were enrolled. High-immunologic-risk KT was defined as one of the following criteria: HLA mismatch number ≥ 5, calculated pretransplant panel reactive antibody ≥30%, pretransplant desensitization performed, positive flow cytometry cross-match, and KT from expanded criteria donor. To date, there are no prospective clinical studies on the ability of individual omics biomarkers to predict acute rejection and no studies on an integrated risk score. Therefore, the number of subjects in this pilot study was not calculated based on clinical evidence, and the number of subjects was determined by considering the incidence of acute rejection in high-risk KT recipients in Korea and the number of high-risk transplants performed per institution. All patients provided written informed consent at enrollment.

**Figure 6:**
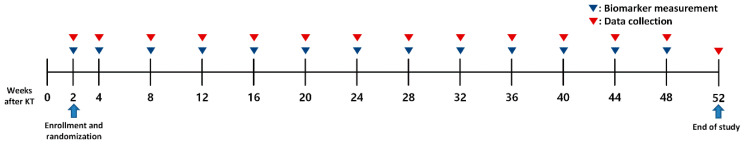
Study design. Abbreviation: KT, kidney transplantation.

The patients were randomly assigned to a biomarker or control group at a ratio of 1:1 using the random number table method by a statistician who was not involved in the study. The random assignment results were immediately reported to the transplant clinicians. The omics biomarkers for AR were measured in blood and urine samples at 2 weeks and every 4 weeks until 1 year after KT (Figure 6). The biomarker group was informed about the measurement results but not the control group. After the biomarker levels stabilized following the initial period of transplantation, the investigators in the biomarker group increased the intensity of immunosuppression if the score increased by more than 1 point on any omics item. During the study period, graft biopsy was performed at the discretion of each transplant clinician, considering clinical findings such as a decrease in eGFR ≥ 30%, spot urine protein-to-creatinine ratio ≥ 0.5 g/g, and BK viremia (>10^4^ copies/mL) or viruria (>10^7^ copies/mL) in both groups. The results of the graft biopsies were interpreted by nephropathologists at each institution. The immunosuppressant dose was adjusted by transplant clinicians considering immunologic and nonimmunologic risks such as infection and nephrotoxicity. The biomarker group also made adjustments to immunosuppression based on the measured integrated risk score: an increase of even one point in each omics item increased the intensity of immunosuppression. In the absence of evidence of AR, the target tacrolimus trough level was set to 5–10 ng/mL, with lower trough levels in specific situations requiring immunosuppressive dose reduction such as BK virus infection. In addition, the mycophenolic acid dose was 1000–2000 mg/day.

### 4.2. Data Collection

The baseline demographic characteristics of the recipient and donor and information on immunosuppressant use were collected at enrollment. In addition, baseline laboratory findings, such as complete blood cell count, sodium, potassium, creatinine, and eGFR levels, were recorded. Serum creatinine, eGFR, and tacrolimus trough levels were measured every 4 weeks. eGFR was calculated using the Chronic Kidney Disease Epidemiology Collaboration equation [25]. Histopathological findings were collected from KTRs who underwent graft biopsy during the study period. AR was diagnosed on the basis of histopathologic findings in accordance with the Banff classification [26].

### 4.3. Omics Biomarkers and Integrated Risk Score

We have identified and validated five transcriptomic and proteomic biomarkers [7,10,11,12,13]. Detailed information on each omics biomarker has been provided in our previous studies. Blood mRNA biomarkers were measured via real-time polymerase chain reaction (PCR) for three genes (*ARG1, MAPK9,* and *OLR1*) [10]. Urinary exosomal microRNA biomarkers were measured via quantitative real-time PCR for three genes (*miR-21-5p, miR-31-5p,* and *miR-4532*) [12]. Furthermore, urinary mRNA biomarkers were measured via quantitative real-time PCR for six genes (*C1QB, CD3e, CXCL-9, IP-10, PSMB9,* and *Tim-3*) [11]. Two urinary exosomal proteomic biomarkers (hemopexin and tetraspanin-1) were measured via Western blotting [7].

A biomarker that exceeded the cutoff value was given 1 point. Hemopexin and tetraspanin-1 were given 1 point each when they reached or exceeded the cutoff value. The integrated risk score was calculated by summing the points for each omics biomarker and ranged from 0 to 5.

### 4.4. Measurement of Omics Biomarkers

At each visit, 2.5 mL of blood and 50 mL of urine were collected. After collection, the blood was stored in PAXgene Blood RNA tubes, and total RNA was extracted using a PAXgene Blood RNA Kit (PreAnalytiX; Qiagen, Hilden, Germany) according to the manufacturer’s instructions. The urine was centrifuged at 2000× *g* for 20 min, the pellet was transferred and stored at −70 °C until use. Total RNA was extracted from the urinary pellets using a PureLink RNA Mini Kit (Invitrogen, Carlsbad, CA, USA) according to the manufacturer’s recommendations. Urinary exosomal RNA was isolated from 1 mL of urinary supernatant using spin column-based exoRNeasy serum/plasma midi kits (QIAGEN GmbH, Hilden, Germany). Then, 20 μg of urinary exosomal proteins was separated by SDS-PAGE, transferred to a nitrocellulose membrane, probed with the appropriate primary antibodies, and incubated with horseradish peroxidase-linked secondary antibodies. Blots were visualized with enhanced chemiluminescence detection reagents and quantified using ECL hyperfilm. Band volumes were measured by densitometry in at least three different experiments. Primary antibodies against the following proteins were used: tetraspanin-1 (H00010103, 1:500; Abnova, Taipei, Taiwan) and hemopexin (ab124935, 1:500; Abcam, Cambridge, UK). The details are shown in our previous studies [7,10,11,12,13].

### 4.5. Outcomes

The primary outcome was eGFR at 1 year after KT. The secondary outcomes were the incidence of AR and graft failure and the frequency of graft biopsy. The tacrolimus trough levels were also compared.

### 4.6. Immunohistochemistry for C4d

For C4d staining, a formalin-fixed, paraffin-embedded kidney tissue was sliced into 2 mm sections and stained with a primary rabbit anti-C4d rabbit monoclonal antibody (Clone SP91; Cell Marque, Rocklin, CA, USA). C4d staining was performed with the BenchMark ULTRA immunohistochemical stainer (Ventana medical system, Tucson, AZ, USA), according to the manufacturer’s protocol.

### 4.7. Statistical Analysis

Continuous variables were expressed as mean ± standard deviation and categorical variables as number and percentage (%). Student’s *t*-test was employed to compare differences between the continuous variables, and Pearson’s chi-squared test or Fisher’s exact test was employed to investigate differences between categorical variables. Statistical analysis was conducted using SPSS version 25.0 (SPSS Inc., Chicago, IL, USA). *p* < 0.05 was considered to indicate statistical significance.

## Figures and Tables

**Table 1:** Baseline characteristics.

	Biomarker (n = 16)	Control (n = 19)	*p*
Age, years	49.4 ± 11.0	52.3 ± 13.4	0.497
Sex, male n, %	12 (75.0)	13 (68.4)	0.668
Body mass index, kg/m^2^	22.0 ± 2.5	21.6 ± 3.5	0.665
Primary renal disease, n (%)			0.260
Diabetes mellitus	6 (37.5)	3 (15.8)	
Hypertension	1 (6.3)	4 (21.1)	
Glomerulonephritis	3 (18.8)	1 (5.3)	
Chronic glomerulonephritis	5 (31.3)	8 (42.1)	
Others	1 (6.3)	3 (15.8)	
Number of HLA mismatch	4.1 ± 1.3	3.7 ± 1.9	0.507
Direct cross-match positive, n (%)	9 (56.3)	7 (36.8)	0.251
CDC-XM positive	5 (31.3)	4 (21.1)	0.700
FCXM positive	7 (43.8)	7 (36.8)	0.678
ABO incompatible, n (%)	5 (31.3)	11 (57.9)	0.115
Pretransplant DSA, n (%)	7 (43.8)	4 (21.1)	0.150
Pretransplant desensitization, n (%)	13 (81.3)	14 (73.7)	0.595
Calculated PRA ≥ 30%, n (%)	2 (12.5)	3 (15.8)	1.000
Comorbid conditions, n (%)			
Diabetes mellitus	7 (43.8)	3 (15.8)	0.132
Hypertension	8 (50.0)	14 (73.7)	0.149
Cardiovascular disease	2 (12.5)	1 (5.3)	0.582
Induction therapy, n (%)			0.142
Basiliximab	7 (43.8)	13 (68.4)	
Anti-thymocyte globulin	9 (56.3)	6 (31.6)	
Laboratory findings at discharge			
White blood cell, ×10^9^/L	9.3 ± 3.5	7.9 ± 3.2	0.218
Hemoglobin, g/dL	10.8 ± 1.1	11.6 ± 1.3	0.065
Platelet, ×10^9^/L	196.7 ± 62.2	188.1 ± 78.1	0.724
Sodium, mEq/L	139.4 ± 2.6	138.3 ± 3.1	0.263
Potassium, mEq/L	4.5 ± 0.5	4.7 ± 0.5	0.225
Creatinine, mg/dL	0.94 ± 0.37	1.14 ± 0.47	0.538
eGFR, mL/min/1.73 m^2^	79.9 ± 29.0	70.5 ± 24.8	0.418
Donor age, years	49.9 ± 9.8	52.6 ± 7.1	0.354
Donor type, n (%)			0.238
Living	13 (81.3)	12 (63.2)	
Deceased	3 (18.8)	7 (36.8)	
KDPI score > 60%	2 (12.5)	7 (36.8)	0.135
KT from expanded criteria donor, n (%)	2 (12.5)	5 (26.3)	0.415

Abbreviations: CDC-XM, complement-dependent cytotoxicity cross-matches; FCXM, flow cytometry cross-matches; DSA, donor-specific antibody; PRA, panel reactive antibodies; eGFR, estimated glomerular filtration rate; KDPI, Kidney Donor Profile Index; KT, kidney transplantation.

**Table 2:** Graft function and biopsy.

	Biomarker (n = 16)	Control (n = 19)	*p*
Scr at 1 year after KT, mg/dL	1.36 ± 0.81	1.37 ± 0.50	0.984
eGFR at 1 year after KT, mL/min/m^2^	55.0 ± 16.0	55.5 ± 18.5	0.946
Scr change (Δ) at 1 year after KT, mg/dL	–0.17 ± 0.61	0.01 ± 0.47	0.452
Scr > 1.5 mg/dL at 1 year after KT	1 (6.3)	6 (31.6)	0.062
Graft biopsy during 1 year after KT, n (%)	2 (12.5)	9 (47.4)	0.027
BPAR during 1 year after KT, n (%)	1 (6.3)	2 (10.5)	1.000
TCMR	1 (6.3)	0	0.457
ABMR	0	2 (10.5)	0.489
Development of dnDSA during 1 year after KT, n (%)	1 (6.3)	2 (10.5)	1.000

Abbreviations: Scr, serum creatinine; KT, kidney transplantation; eGFR, estimated glomerular filtration rate; BPAR, biopsy, biopsy-proven acute rejection; TCMR, T-cell-mediated rejection, ABMR, antibody-mediated rejection; dnDSA, de novo donor-specific antibody.

**Table 3:** Histopathological findings of biopsy in patients with diagnosed rejection.

Patient No.	Diagnosis	t	i	g	ptc	MVI	v	C4d	ci	ct
B-1	Acute TCMR, IB	3	2	1	2	3	0	0	0	0
C-1	Active ABMR	0	0	0	2	2	0	1	0	0
C-2	Active ABMR	1	2	2	2	4	0	0	1	1

Abbreviations: TCMR, T-cell-mediated rejection; ABMR, antibody-mediated rejection.

## Data Availability

All relevant data are within the paper and its Supplementary Materials.

## Supplementary Materials

The following supporting information can be downloaded at: https://www.mdpi.com/article/10.3390/ijms25105139/s1.
