# Supplementary figures and images for: Efficacy of Integrated Risk Score Using Omics-Based Biomarkers for the Prediction of Acute Rejection in Kidney Transplantation: A Randomized Prospective Pilot Study
Source: Int J Mol Sci. 2024 May 9;25(10):5139. doi: 10.3390/ijms25105139 (PMC11121528; doi:10.3390/ijms25105139)

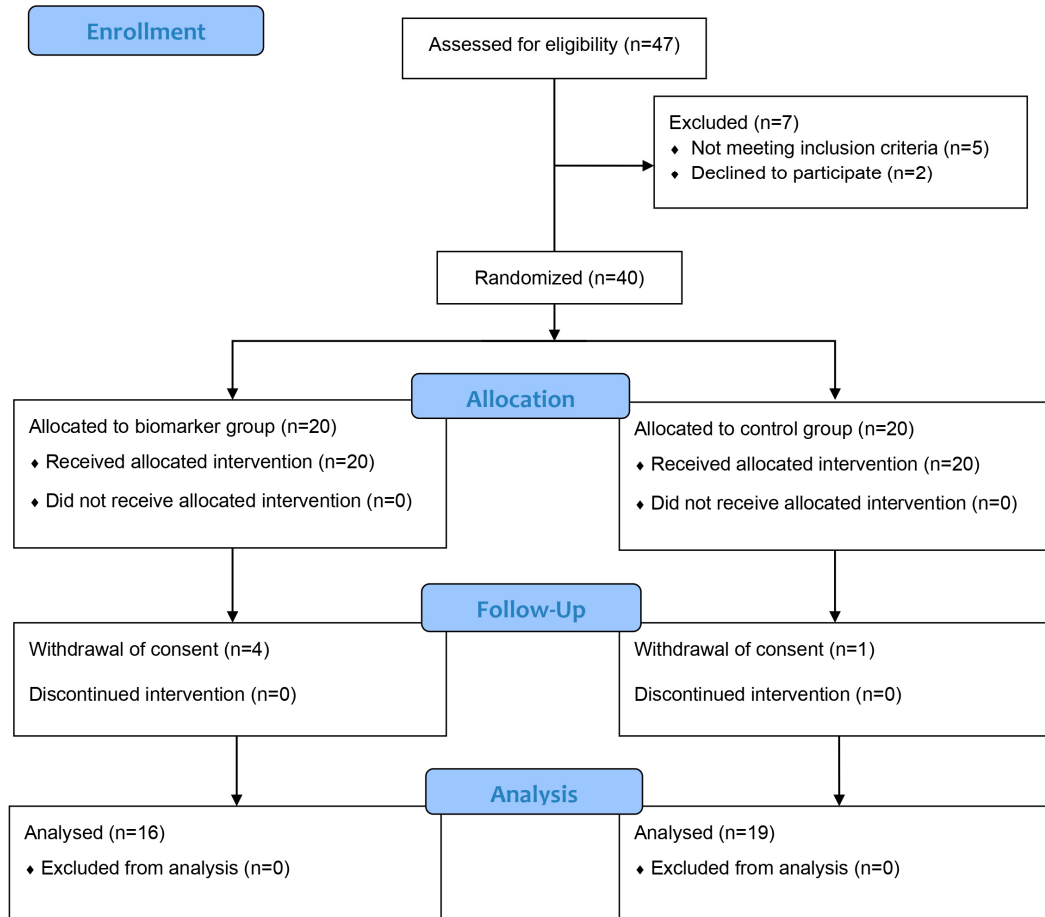

Figure S1. Flow diagram of the study.

Supplement: Supplementary file 1 [file ijms-25-05139-s001.zip › FIgure S1.pdf]
